# The Great Yellow Peril

**Published:** 1916-09

**Authors:** Charles H. Duncan

**Affiliations:** New York, N. Y.


### The Great Yellow Peril.

(Continued from August issue.)

BY CHARLES H. DUNCAN, M. D., NEW YORK, N. Y.

### MEDICAL PREPAREDNESS.

*Autosepsis* gives us more latitude in our operative surgical technic and is a God-send to the army surgeon and soldiers; correcting faulty surgical aseptic technic. It is only a question of time before it will be compulsory on the part of the army surgeons to treat their patients autotherapeutically and to teach the soldiers by means of printed cards how he may so treat himself before seen by the surgeon, that infection may be aborted. *When we are able to obtain the infecting micro-organisms in the exudate, and use them autotherapeutically, there is no necessity for a wound ever to become purulent.* The army hospital method for aborting infection is as follows: Cleanse the wound with only normal salt solution and dress it daily with sterile gauze, if it can be obtained. At each dressing catch all of the oozing from the wound that is possible, using the stained gauze that is immediately over the wound and the drain if there is one. Add enough distilled water to bring it to an ounce of fluid. The gauze and water are placed in a bottle and well shaken. It is allowed to stand for from twelve to twenty-four hours. At the end of this time it is filtered through a Duncan autotherapeutic apparatus and from 1 to 4 c.c. injected hypodermatically in the loose cellular tissues over the biceps muscle; keep this treatment up daily till the danger of infection is passed. A method of hurrying this process consists of grinding one c.c. of the discharge in a mortar with powdered glass; after it is thoroughly ground it is placed in an ounce bottle of water, shaken thoroughly to dissolve the soluble toxins. It is then allowed to stand for an hour or two, after which time it is filtered and injected in the manner described. Allowing the mixture of water and discharge to stand, and the agitation as part of the first process as well as the grinding of the second, destroys some of the micro-organisms mechanically. Their toxins will then go into solution by autolysis and be in the filtrate ready for use. This is the universal autotherapeutic technic for all wounds, but it is not necessary to employ this technic in the prevention of infection in extra-alimentary and extra-pulmonary wounds, as the convenient oral method may be used in wounds of these parts.

The technic of the application of the autotherapeutic principle in the abortion of infection in extra-alimentary and extra-pulmonary wounds that is recommended to the army surgeons is as follows: Dress the wound twice daily. At each dressing cut out about four inches of the stained gauze immediately over the wound, place it in a four-ounce bottle of water, shake well, squeeze out the gauze and give the decanted fluid to the patient to drink at once. I recommend the last procedure highly for its simplicity, convenience and great therapeutic value. The first inoculating micro-organisms are few in number; they must multiply extensively before pus can be seen. At the time this treatment is being carried out, the system of the young soldier, who is usually strong and robust, is better able to react the toxins than it would be later, for it is not then reduced with the fever, coincident with pyogenic infections. The micro-organisms are not as virulent then as later when many successive generations of them have developed. If we place a toxin developed in a turtle, guinea pig, rabbit or a test-tube, or a toxin from some other patient or source, in comparative healthly tissues, these tend to develop resistance or reaction opposite or specific to these toxins, respectively, but this is not the reaction to the toxin from which the patient suffers, and the resulting cure is wholly problematical. To give any other than the patient's own unmodified toxin is guesswork prescribing, pure and simple and wholly unscientific, for there is no certainty that any heterogeneous toxin will develop a curative reaction in any given patient; experience for the past century has proven this. The patient is suffering from his own unmodified toxins that have developed in his own body from the activity of both causative and complicating micro-organisms, and from a set of toxic tissue substances, as enzymes, ferments and toxic results of chemical changes in the protoplasma molecules that correspond to each bacterial toxin, respectively, against which the tissues react in a curative manner. When this unmodified parent toxin complex is placed in comparative healthy tissues the resistance or reaction developed to them is exactly antagonistic, and is the specific resistance to the same toxins remaining in the patient's body.

This treatment is distinctly new and original and entirely foreign to all modern methods of wound treatment, yet wounds will remain free from pus when it is properly given. Free drainage and autoseptic technic will cause almost any non-fatal purulent wound to heal quickly.

The autoseptic method of wound treatment interferes in no way with the established aseptic method, but offers an additional and effective safeguard against infection. In further substantiating my claim to be able to abate aspesis by placing the fresh wound or the discharge from the fresh wound in the mouth, I visited the dog-catching station in New York City. It is believed that the bite of any animal is liable to result in purulent infection and possibly tetanus and hydrophobia, and any neglect to thoroughly cauterize the bite from an animal invites infection. These dog catchers never have purulent infection, tetanus or hydrophobia following their bites when they are able to get the wound in the mouth, for they always suck it the first thing when bitten. The question has been asked so frequently that, since pus by the mouth is efficacious in extra-alimentary and extra-pulmonary wounds, "Why do not patients suffering with tuberculosis or chronic bronchitis cure themselves?" that I will take this means of answering this question and giving what I believe to be the reasons why they do not. Healthy tissues develop maximum resistance or antibodies to toxins placed in them. It appears from clinical experience that tissues involved with disease toxins react to them in reverse ratio to their involvement; that is, the greater the involvement the less the reaction, and, conversely, the less the involvement the greater the reaction they are capable of developing. In stab wounds of the lung it is altogether probable the wound is or becomes infected, for the lungs contain myriads of pathogenic micro-organisms. The wound being infected could be classed as a bronchial or respiratory disease. In respiratory infections there is very little reaction by the tisues of the stomach because the patient is constantly swallowing the toxin contained in the sputum. These tissues are already involved with these toxins, but when the sputum or the discharge from a wound of the lung is filtered through a standard porous filter, such as is employed in connection with the Duncan autotherapeutic apparatus, and the filtrate injected hypodermatically, the cure in all acute conditions is expected quickly. Many chronic infections of the respiratory system are benefited more by this treatment than by any other means.

*Purulent Infection.*—The autotherapeutic technic employed in curing the following infections is given under the case reports. It will readily be seen that the technic varies within wide limits; the basal consideration is that the toxins should be fresh and unmodified by the pathologist; that is to say, the toxins should be as nearly like the natural human unmodified parent toxins developed in the patient's body as it is possible for them to be. The therapeutic value appears to decrease in direct ratio to the laboratory process or manipulation which they undergo during their preparation at the hands of the pathologist. There is no process in the laboratory that adds to their therapeutic value; every step in the process through which they pass alters or detracts from their curative properties. If Wright's vaccine cures, such cure is not because of the laboratory manipulation, but in spite of it. The cases cited have been taken from a list of hundreds that have been treated by myself and by many other physicians scattered throughout the world. If the following technic is closely adhered to, you will have little trouble and often be amazed at the quick cures you have made. This specific medication in purulent infection is the entering wedge that has revolutionized our ideas of medicine.

No. 1.—Autotherapeutic technic for purulent infections.

Fresh pus ........................... minims 6
Distilled water ......................... ounce 1

Sig.: Mix in a bottle, shake well, and allow to stand for twenty-four hours. Filter through a Duncan autotherapeutic apparatus. Inject 20 minims of the bacteria-free filtrate into the loose cellular tissues over the biceps muscle.

There are various modifications of this treatment that are useful at times, but the therapeutic value of none of these has been proved to be greater than that given above. For example, I use the following method in treating desperate cases in which it is necessary to hurry medication. This treatment is useful also when it is impossible to see the patient again. It is useful only because it saves time. The interval between obtaining the pus and giving injection may be materially shortened by thoroughly grinding the six drops of pus in a mortar with powdered glass or fine sharp clean sand previous to mixing with the water. When this is done the mixture should be thoroughly agitated in a bottle with an ounce of distilled water to dissolve the soluble toxins. When the micro-organisms are destroyed their toxins will tend to go into solution by autolysis. The fluid is then allowed to stand for six hours, then filtered through a Duncan autotherapeutic apparatus, and 20 minims are injected at once.

The technic embodied in No. 1 and No. 2 formulas are *universally applicable to all infected wounds.* The technic embodied in

No. 3 and No. 4, also No. 5, formulas is applicable to the largest class of wounds with which we deal; namely, to extra-alimentary and extra-pulmonary infections.

No. 3.—This method of treating a patient suffering with purulent infection is one which the writer has used successfully in treating many thousands of cases, and which he prefers to all others. This preparation will keep, and if no additional microorganism creeps into the wound during the daily dressings it may be used till the case has cleared up.

| | |
|---|---|
| Pus | minims 3 |
| Light curettage from side of wound | minims 3 |
| Milk sugar | ounce 1 |

Sig.: Mix in a mortar and grind thoroughly for ten minutes. Dose twenty grains by the mouth every hour for six doses, then stop medication.

No. 4.—Another method of treatment may be useful when a large number of patients are to be treated within a limited period of time: Place two to six drops of pus on a lump of sugar, or in a little water (colored with cocoa if necessary to disguise any blood), and give to the patient to drink in one dose. Many veterinarians just catch the pus in a spoon, or on a flat stick, and place it on the animal's tongue. They are unanimous in vouching for the specificity of this treatment; their leading men say it would be a crime not to give it to horses.

No. 5.—Another method that may be useful at times is simply to lick the pus from the infected area. If the infection is very recent, this may be done at frequent intervals; if the infection is chronic, this treatment should not be so frequent. When another dose is needed, there is usually some indication for it in the wound itself. It feels irritated, or the patient's attention is attracted to it. If it is then placed in the mouth and licked, the irritation will soon subside. If it is impossible to reach the wound with the tongue, a part of the stained dressing may be chewed. In chronic cases, one dose is often all that is required.

It is desirable that the patient exercise but little during the reactive period of the medication. To open the bowels with calomel and follow with a saline cathartic when the patient is inclined to be constipated is a procedure the writer has found to be useful. If a chill following a hypodermic injection should be pronounced, the patient should rest, cover well, and take a hot drink. No antiseptic should contaminate the discharge that is to be used autotherapeutically. The autotherapy remedy is the only strictly autogenous therapeutic agent we have at our command in fighting disease and death.

If the best results are to be obtained in giving the autotherapeutic medication, the patient should be watched carefully for any change in his condition. Except in very acute cases, no further dose than that mentioned above is given till the patient ceases to improve under the preceding dose. In chronic cases this will often be from the third to the seventh day. In from six to ten hours after the injection, the cutaneous action will be from the size of a silver dollar to that of the palm of the hand. The constitutional reaction is usually slight, the temperature seldom rises above 100° F. After twenty-four hours the cutaneous and constitutional reactions will begin to subside; in forty-eight hours they usually disappear. The pains in the wound will most often cease in from one to twelve hours after the toxins are given. In twenty-four hours the discharge will usually be noticeably less. Coincident with the discharge becoming less, it will become thin and sanguinous, and the clinical symptoms will subside. A thin discharge is an indication that the curative reaction is continuing. No further dose is given as long as the discharge is thin. If the discharge again becomes thick, another dose freshly made is given. Watch your patient carefully, let him be the guide as to when another dose is needed. No set rules will fit all cases. The doses given are for a strong, healthy man. Never use antiseptics on a wound treated autotherapeutically, for many antiseptics destroy the therapeutic value of the toxins; that is, pus containing some antiseptics is useless for autotherapeutic purposes.

(To be continued in November issue.)

